# Supplementary figures and images for: c-Myc plays a key role in IFN-γ-induced persistence of Chlamydia trachomatis
Source: eLife. 2022 Sep 26;11:e76721. doi: 10.7554/eLife.76721 (PMC9512400; doi:10.7554/eLife.76721)

**Figure 1 B**

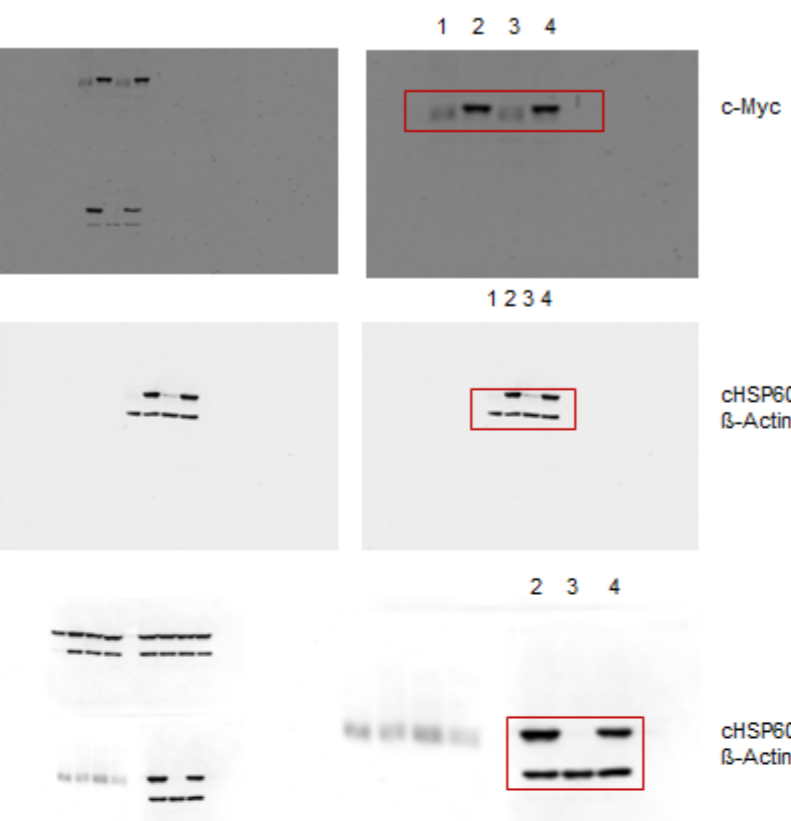

**Figure 1 D**

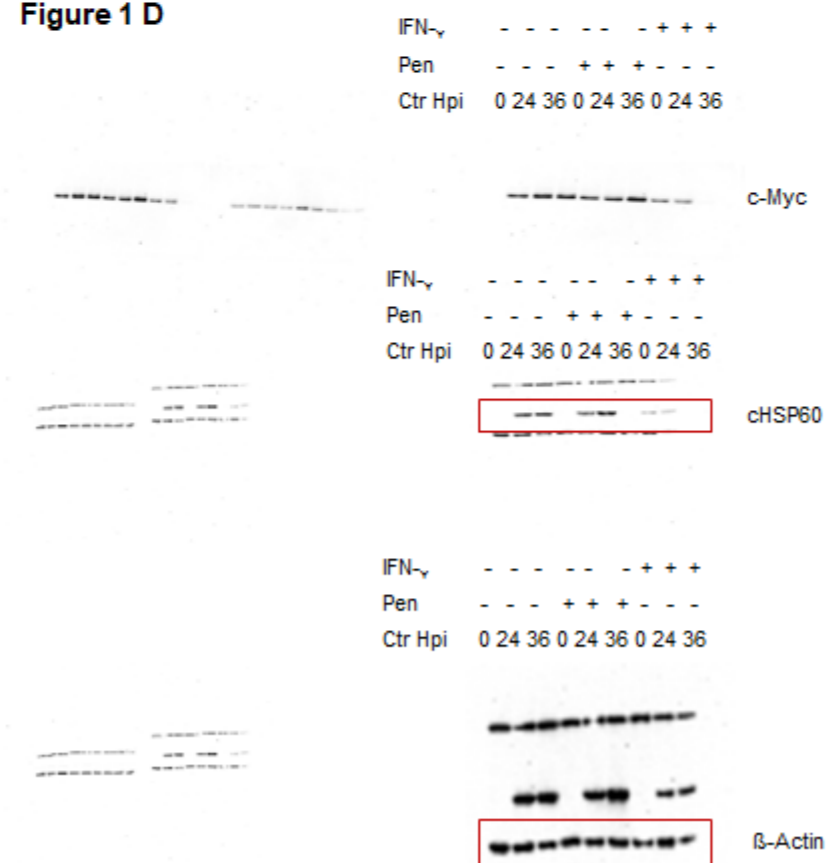

**Figure 1 E**

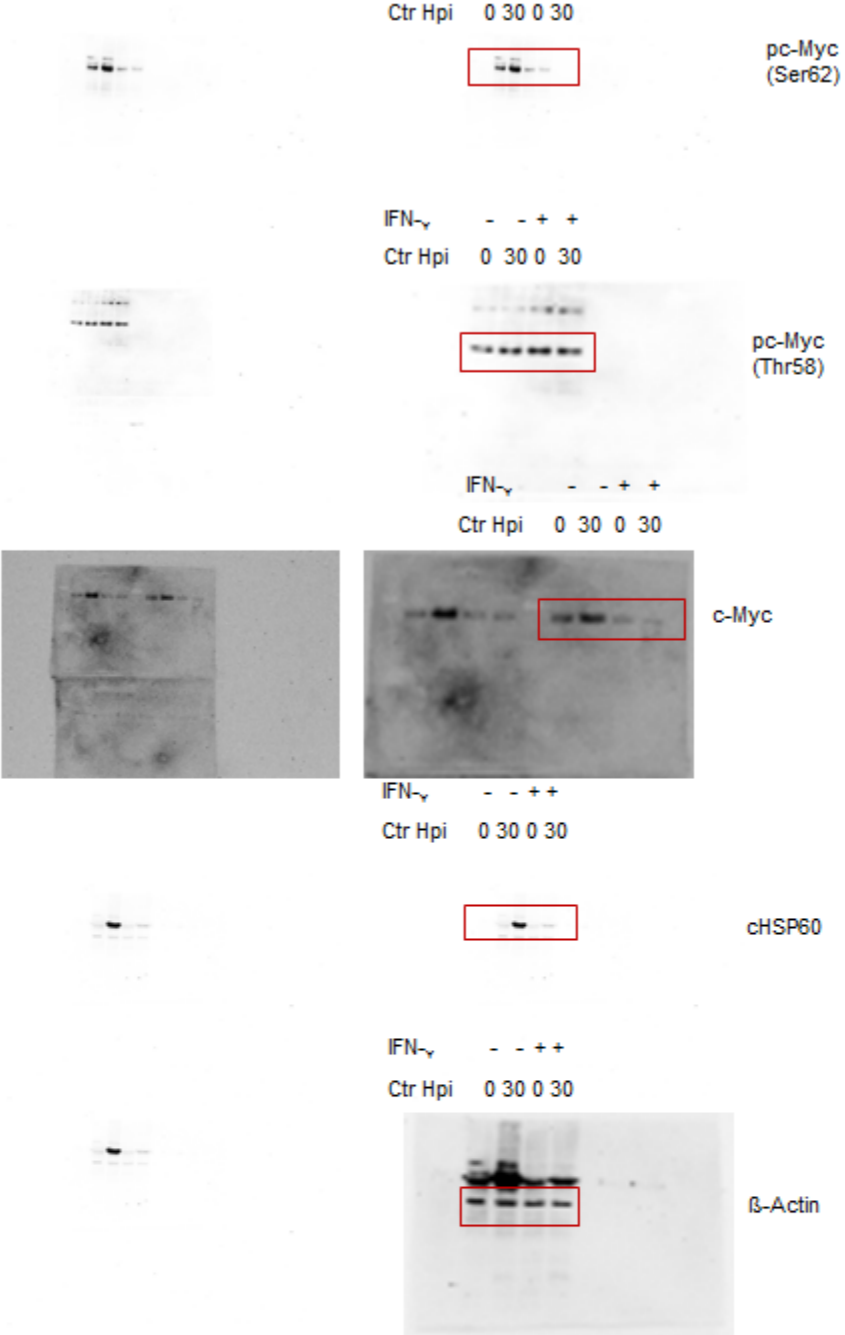

**Figure 1 J**

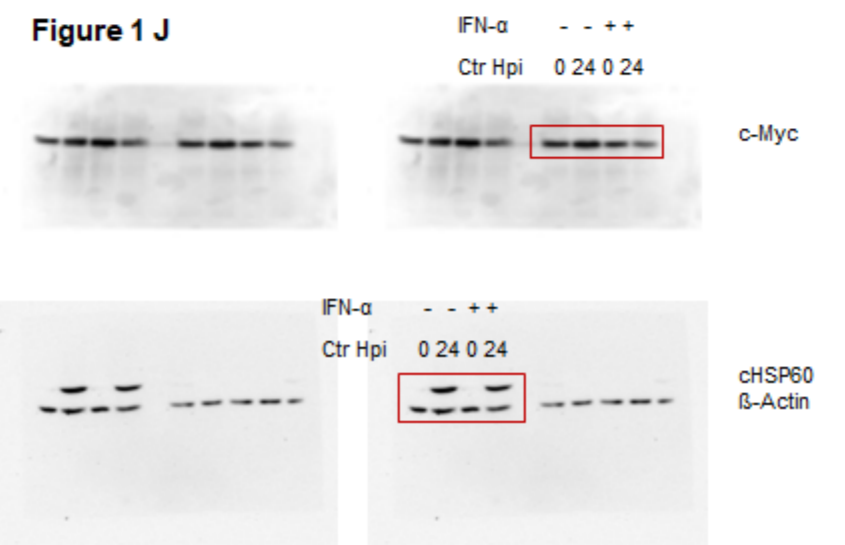

**Figure 1 H**

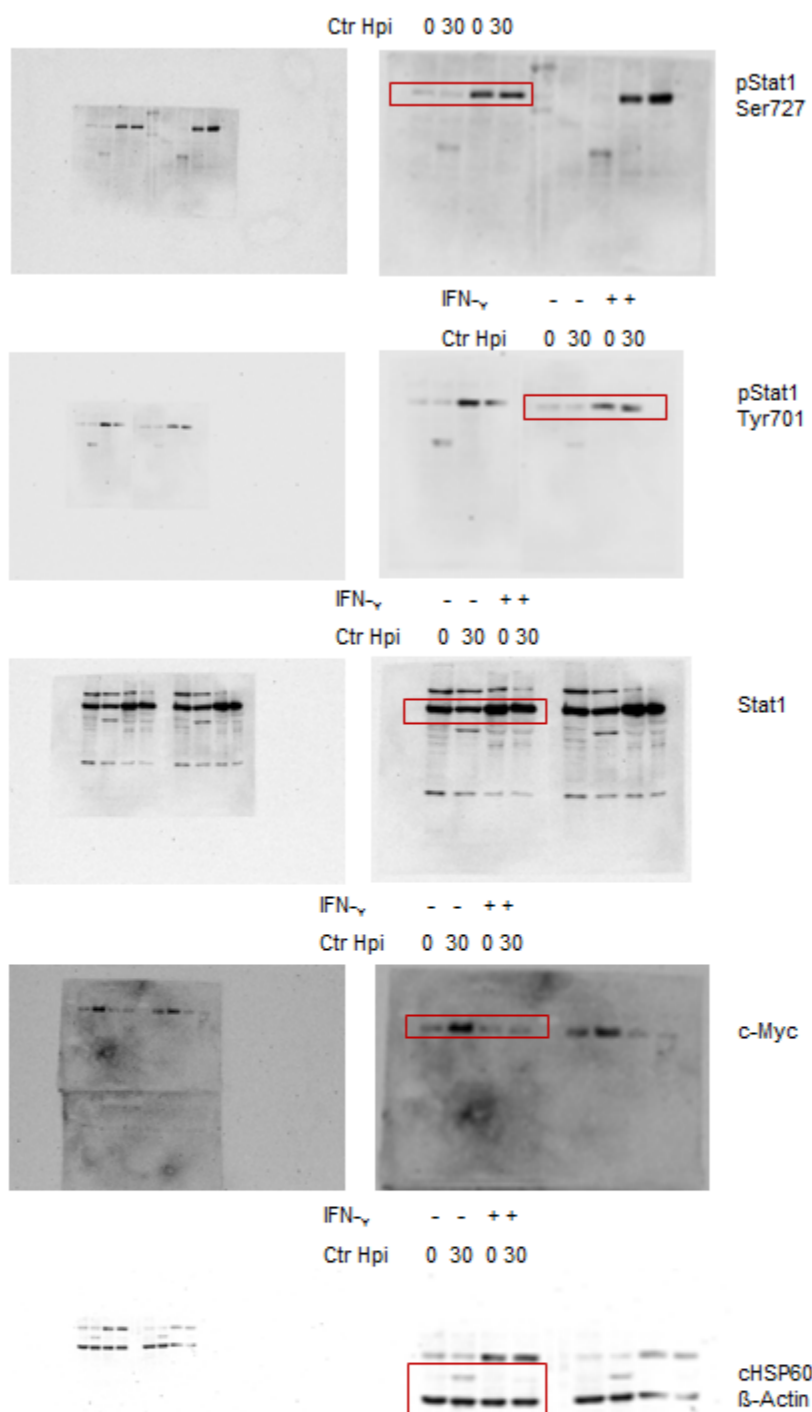

**Figure 1 I**

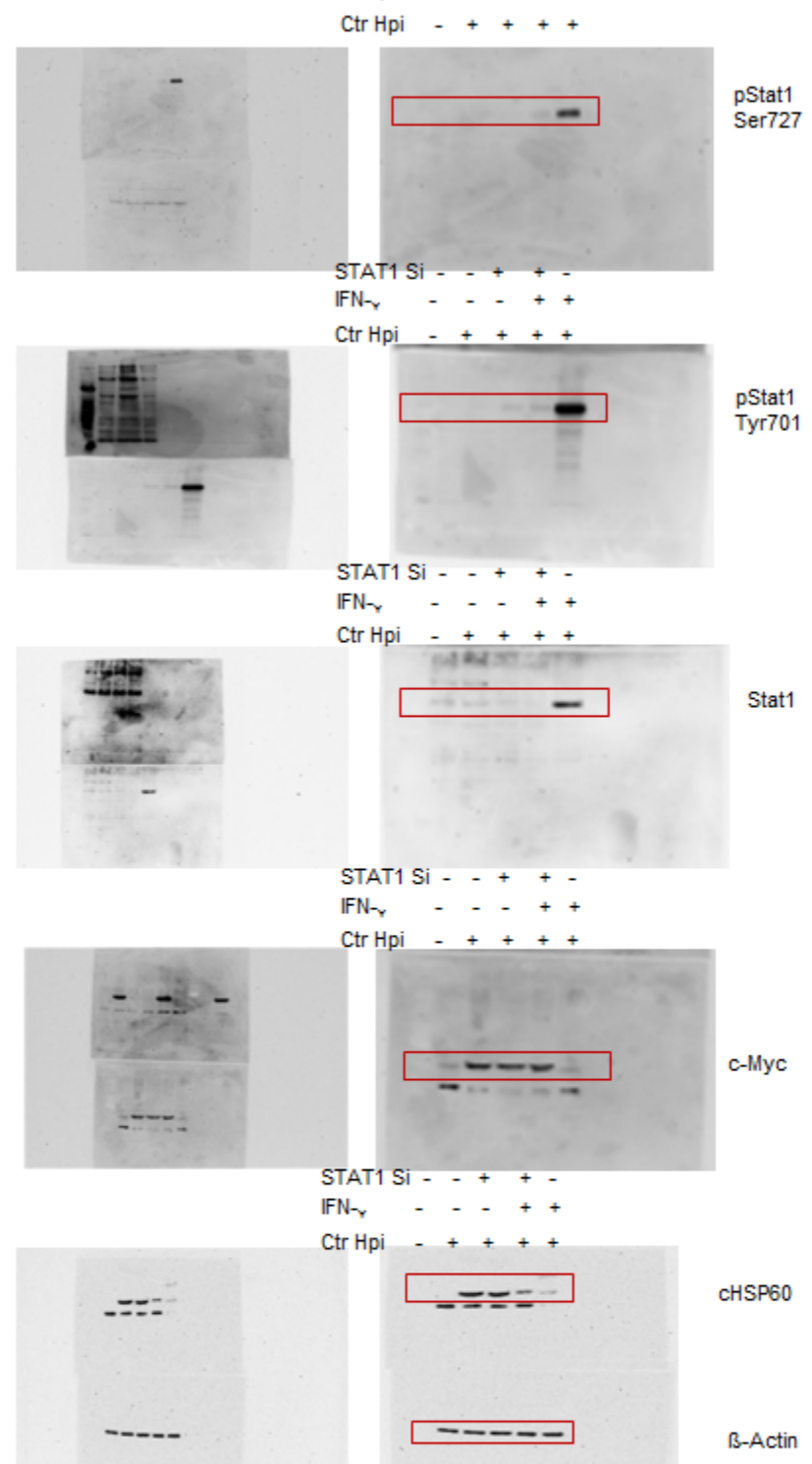

Supplement: Figure 1—source data 1. [file elife-76721-fig1-data1.pdf]

**Figure 2 E**

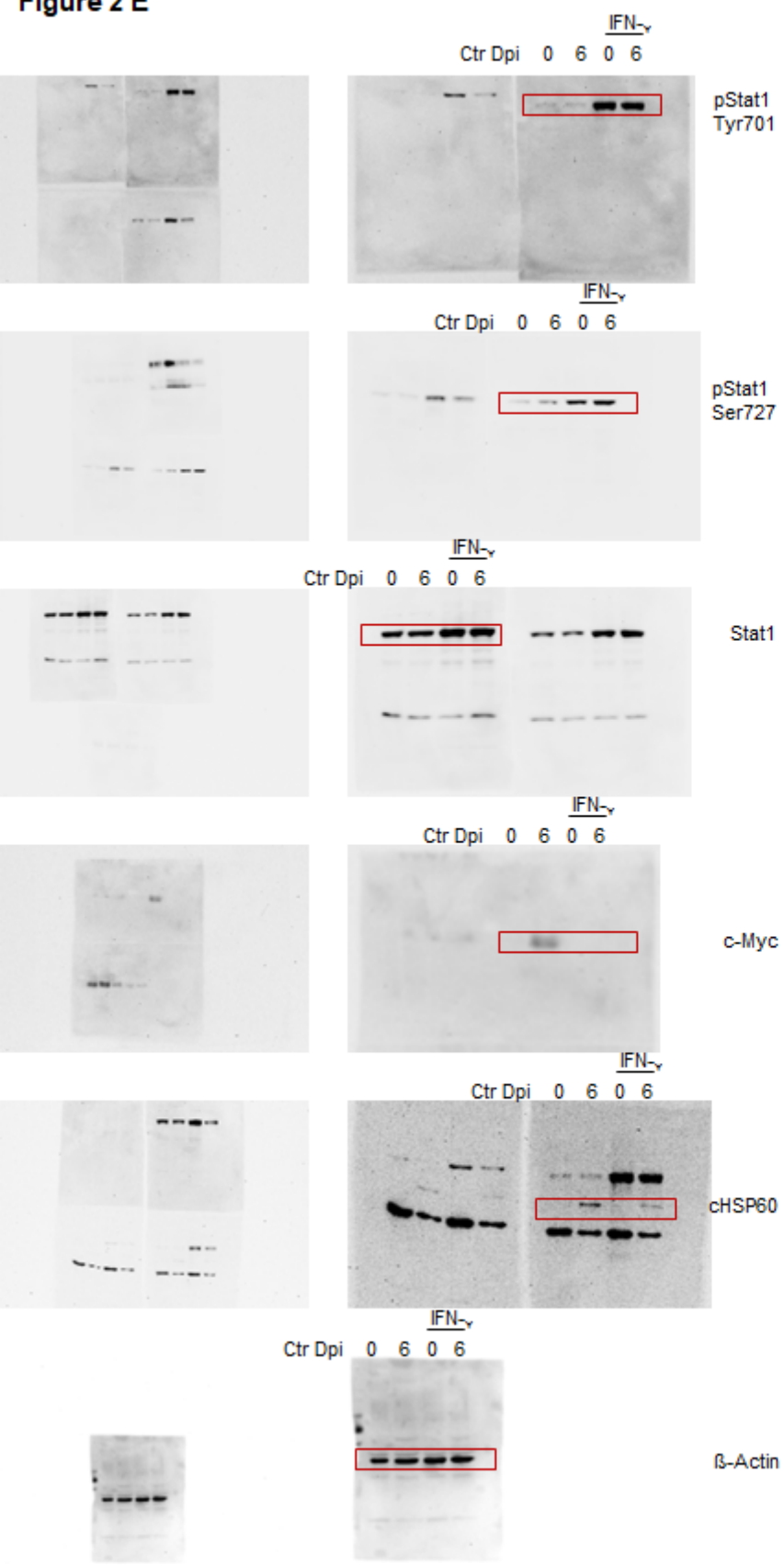

Supplement: Figure 2—source data 1. [file elife-76721-fig2-data1.pdf]

**Figure 3 A**

|               |   |    |    |    |    |    |    |
|---------------|---|----|----|----|----|----|----|
| IFN- $\gamma$ | - | -  | -  | -  | ++ | ++ | ++ |
| AHT           | - | -  | ++ | ++ | -  | -  | ++ |
| Ctr Hpi       | 0 | 30 | 0  | 30 | 0  | 30 | 0  |

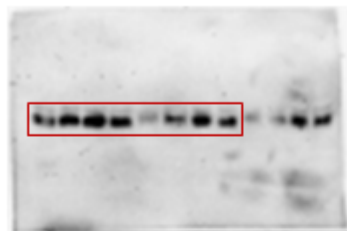

c-Myc

|               |   |    |    |    |    |    |    |
|---------------|---|----|----|----|----|----|----|
| IFN- $\gamma$ | - | -  | -  | -  | ++ | ++ | ++ |
| AHT           | - | -  | ++ | ++ | -  | -  | ++ |
| Ctr Hpi       | 0 | 30 | 0  | 30 | 0  | 30 | 0  |

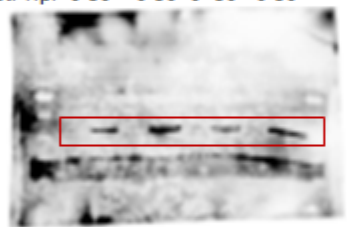

OmpA

|               |   |    |    |    |    |    |    |
|---------------|---|----|----|----|----|----|----|
| IFN- $\gamma$ | - | -  | -  | -  | ++ | ++ | ++ |
| AHT           | - | -  | ++ | ++ | -  | -  | ++ |
| Ctr Hpi       | 0 | 30 | 0  | 30 | 0  | 30 | 0  |

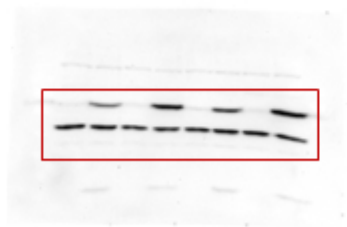

cHSP60  
 $\beta$ -Actin

Supplement: Figure 3—source data 1. [file elife-76721-fig3-data1.pdf]

**Figure 4 A**

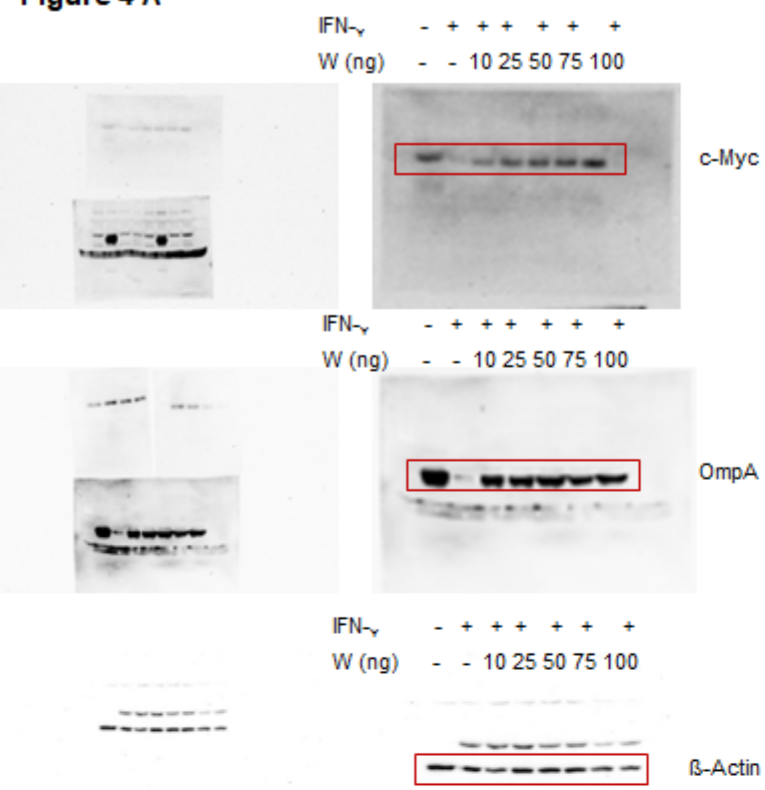

**Figure 4 B**

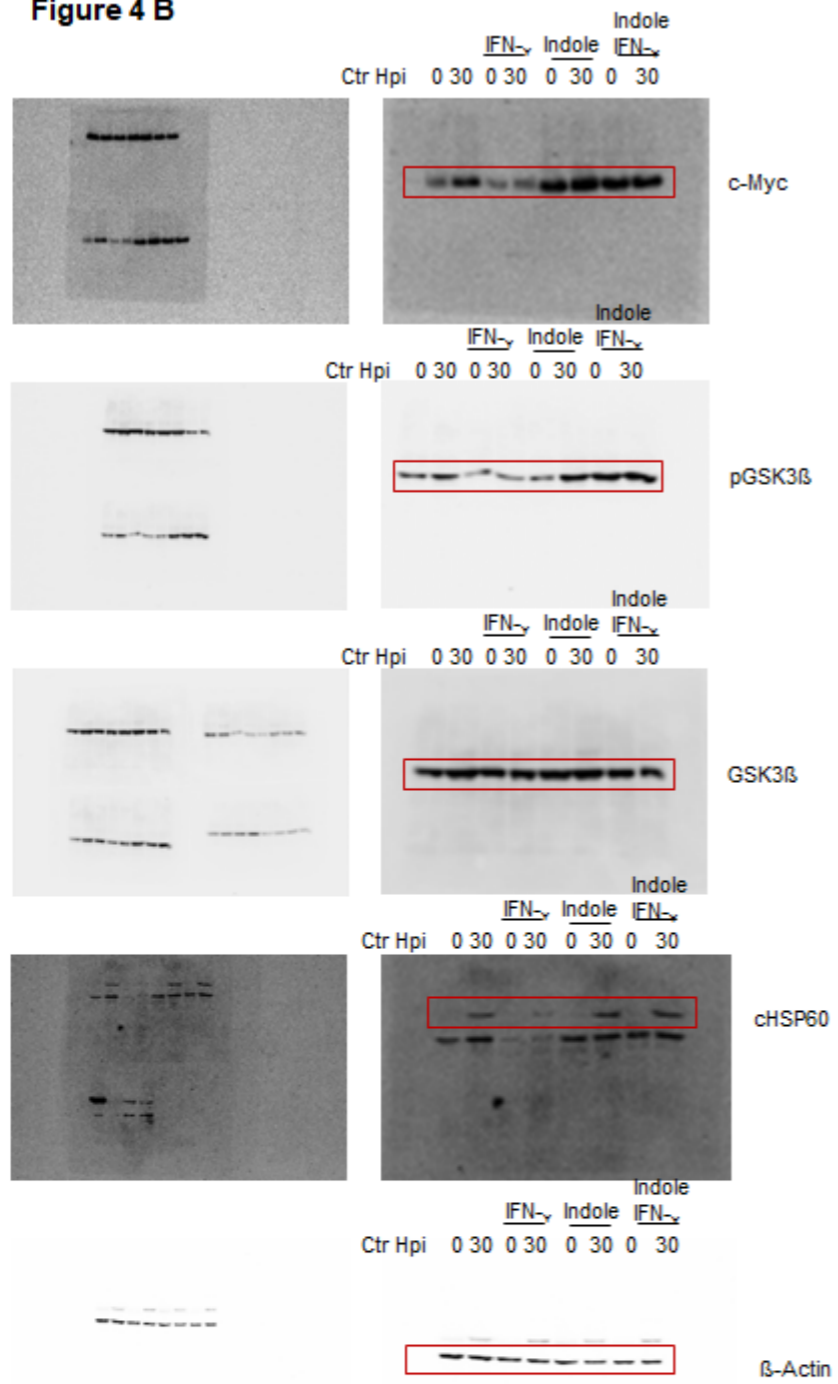

**Figure 4 C**

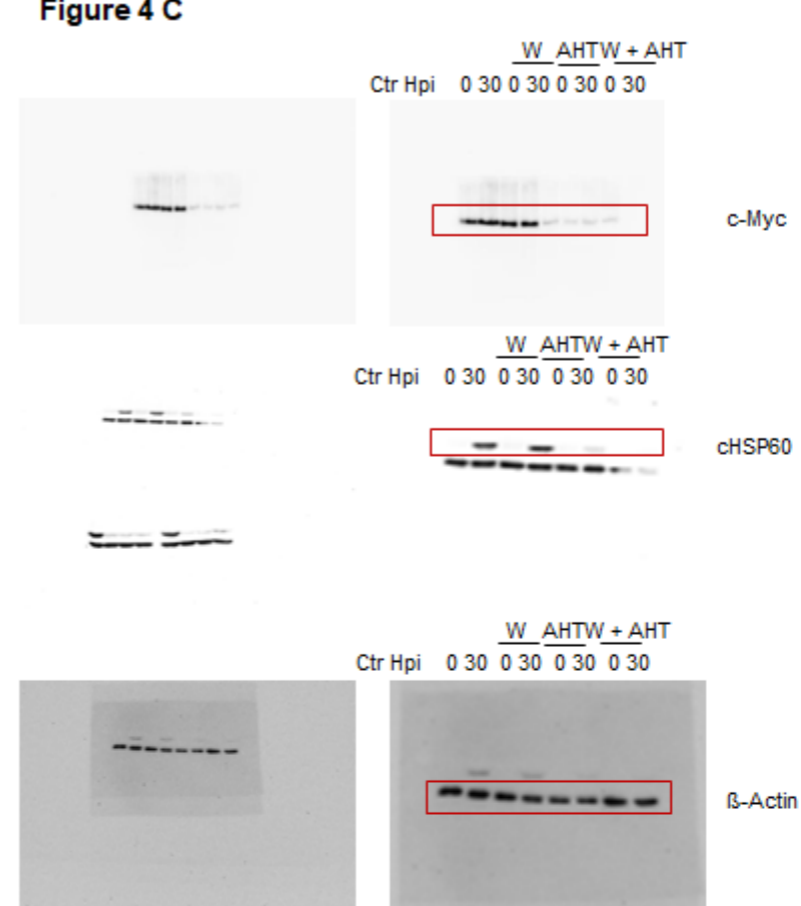

**Figure 4 E**

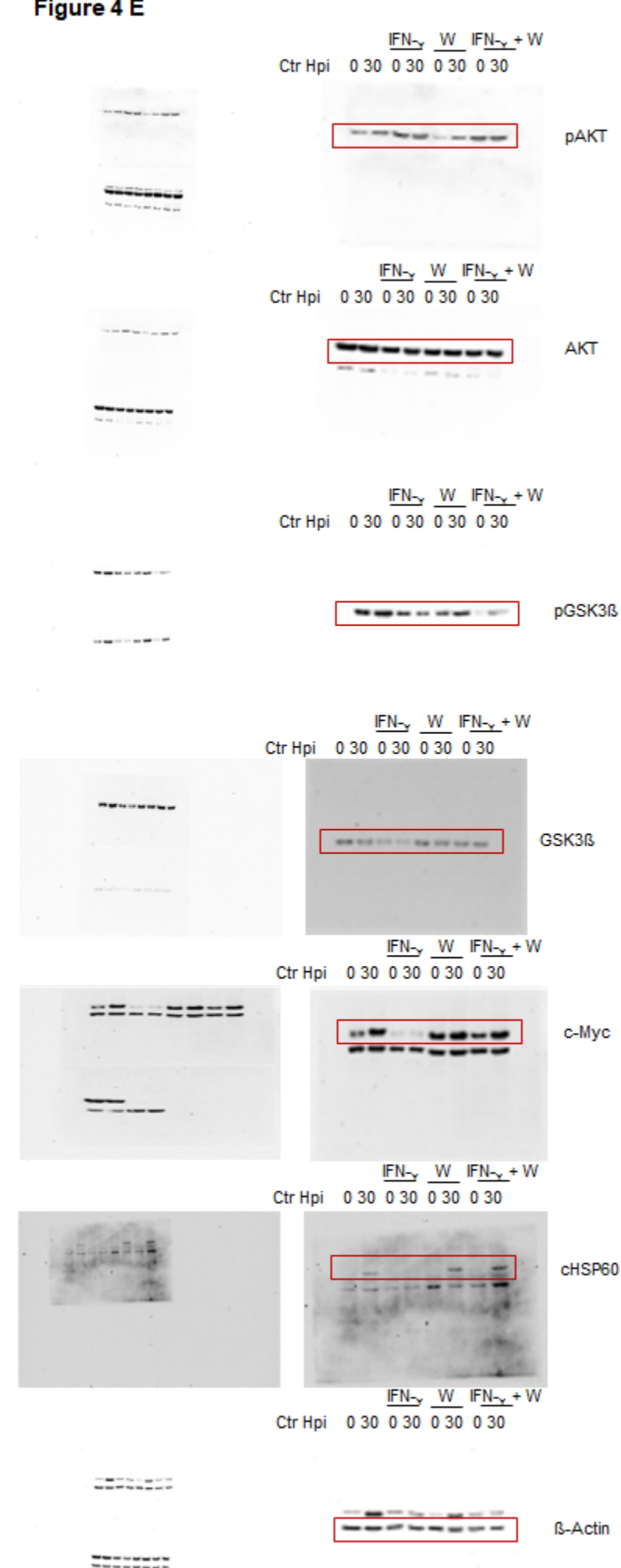

Supplement: Figure 4—source data 1. [file elife-76721-fig4-data1.pdf]

**Figure 5 A**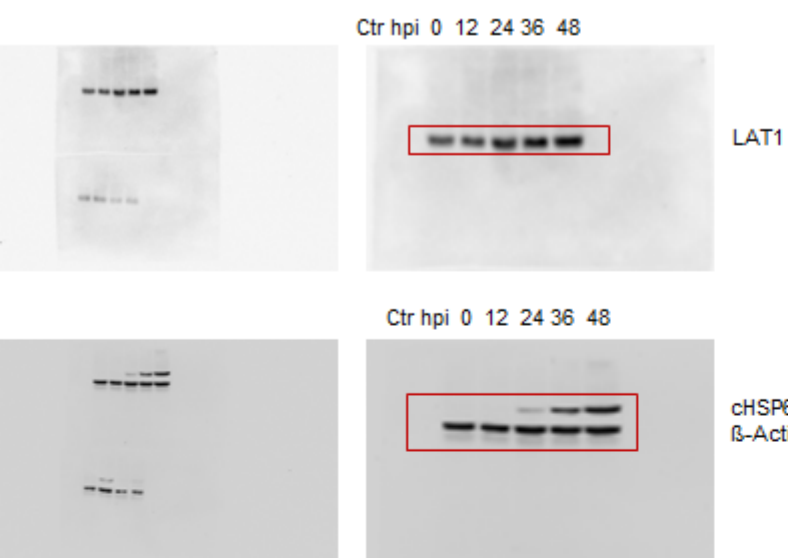**Figure 5 B**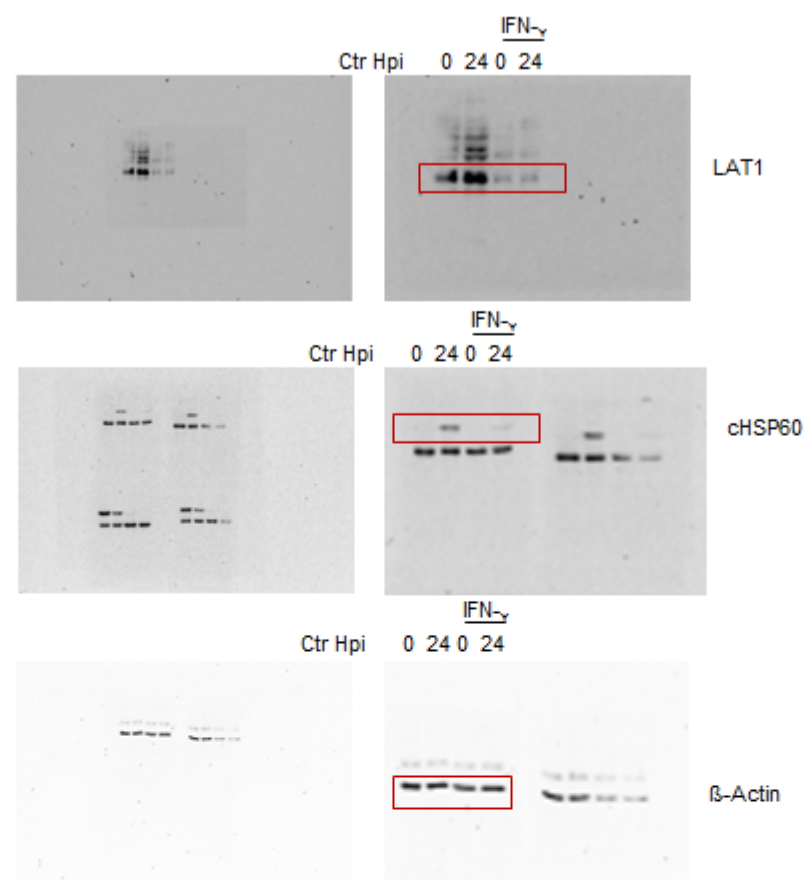**Figure 5 C**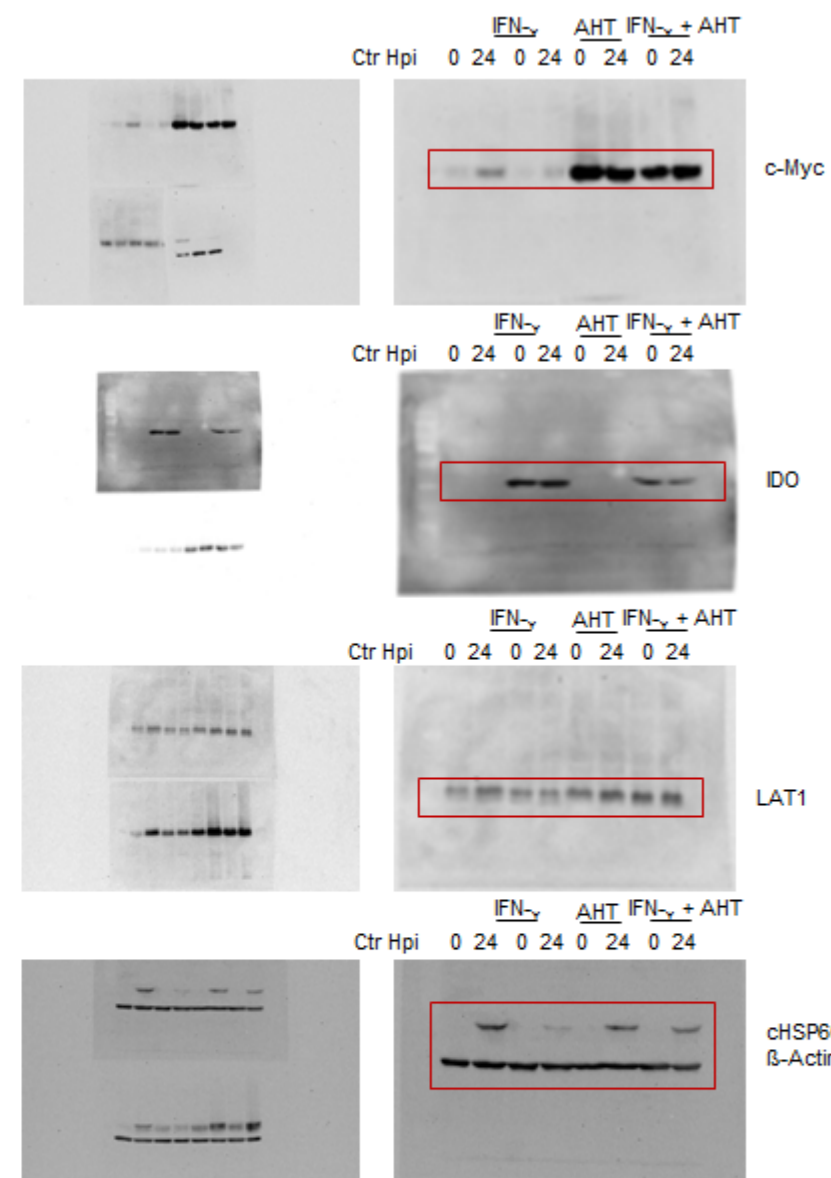**Figure 5 D**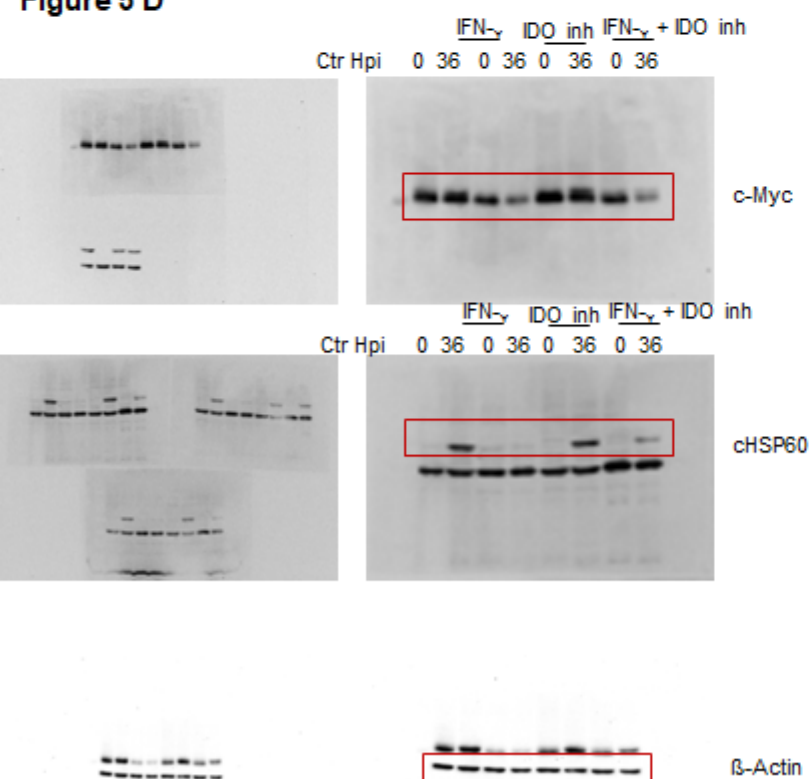**Figure 5 E**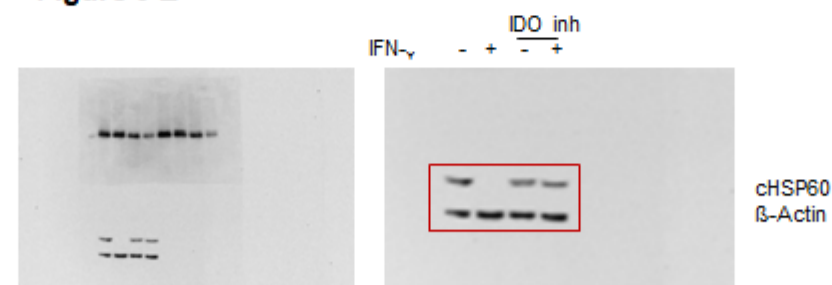

Supplement: Figure 5—source data 1. [file elife-76721-fig5-data1.pdf]

**Figure 6 D**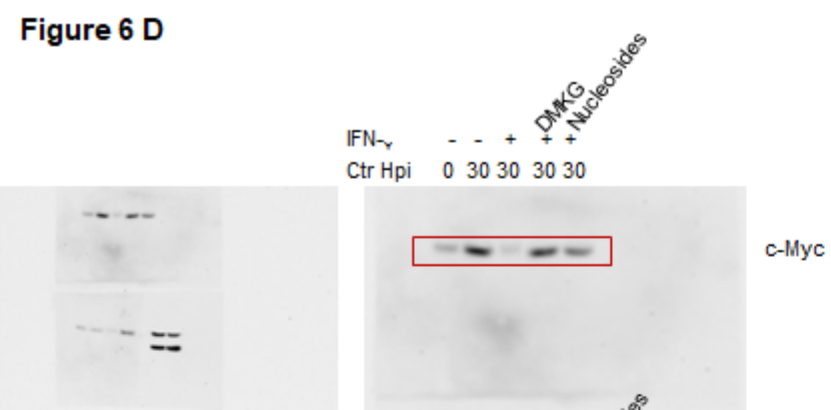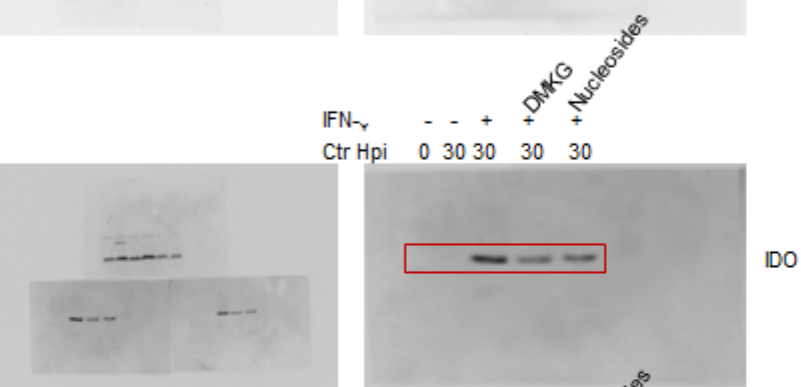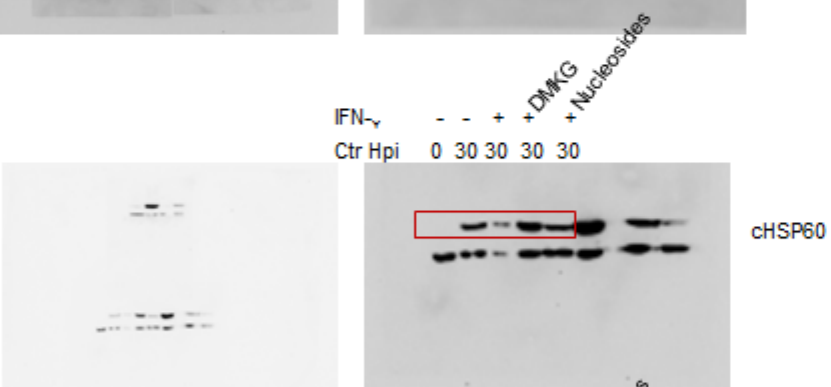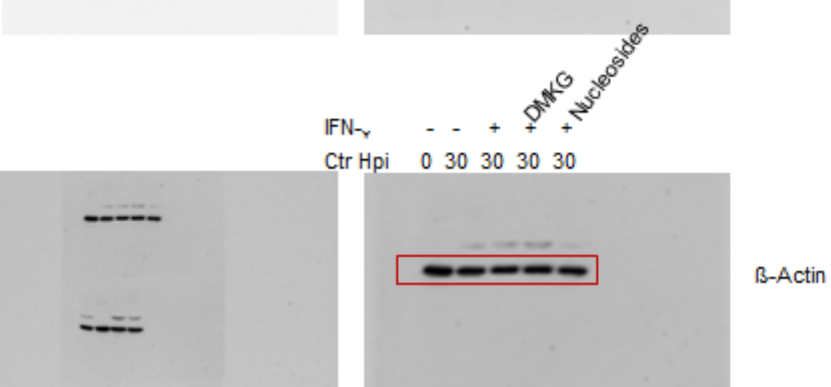**Figure 6 E**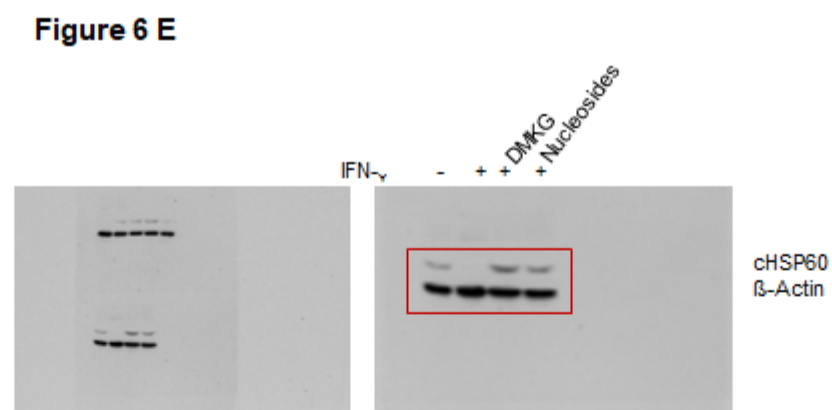**Figure 6 G**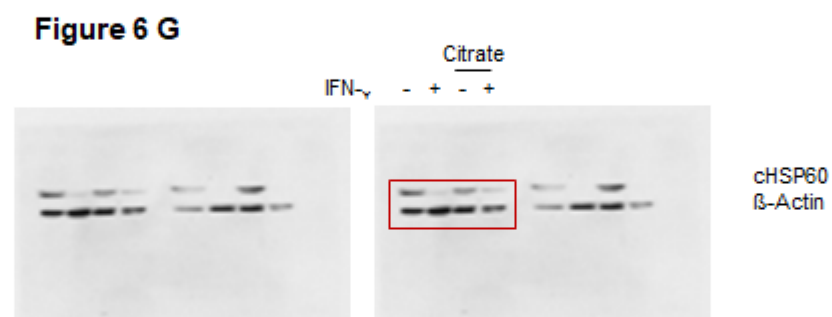**Figure 6 F**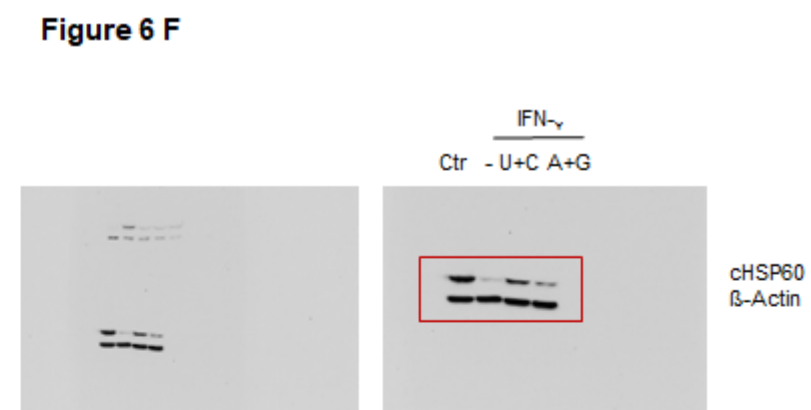**Figure 6 H**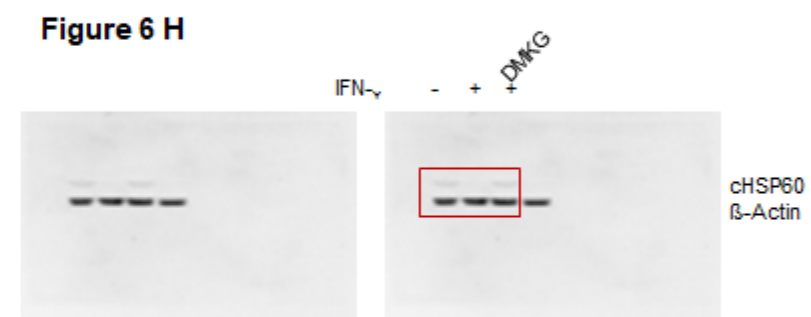

Supplement: Figure 6—source data 1. [file elife-76721-fig6-data1.pdf]

**Figure 7 A**

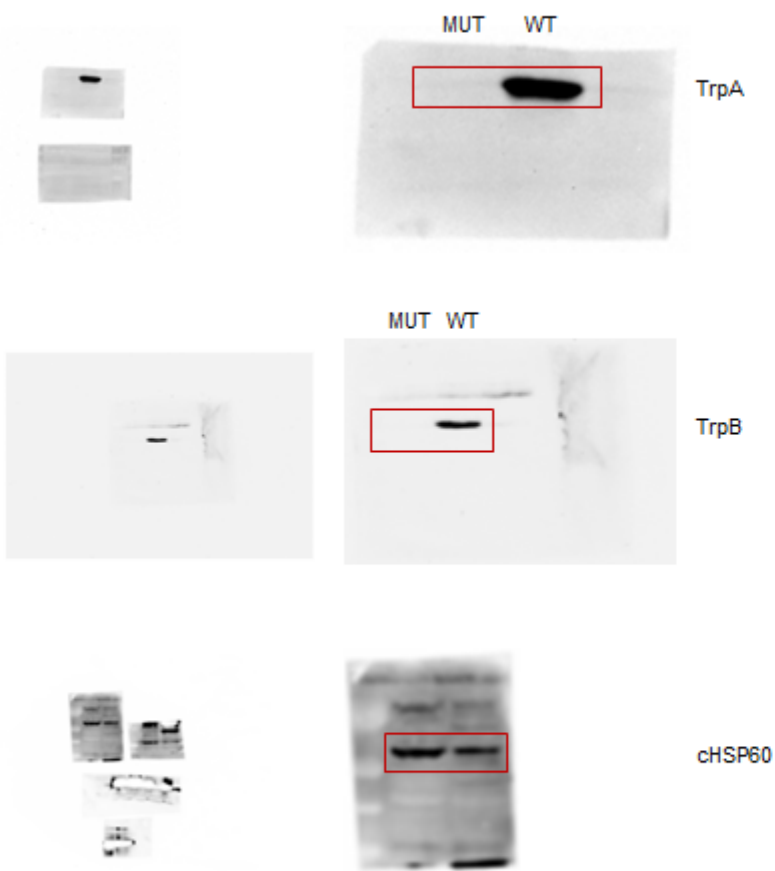

Supplement: Figure 7—source data 1. [file elife-76721-fig7-data1.pdf]
