# Supplementary material for: c-Myc plays a key role in IFN-γ-induced persistence of Chlamydia trachomatis
Source: eLife. 2022 Sep 26;11:e76721. doi: 10.7554/eLife.76721 (PMC9512400; doi:10.7554/eLife.76721)

Figure 1 – figure supplement 1 C

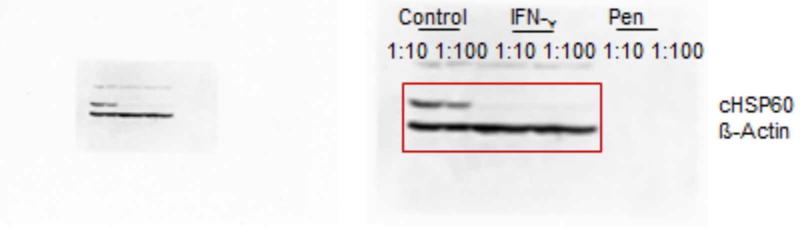

Figure 1 – figure supplement 1 E

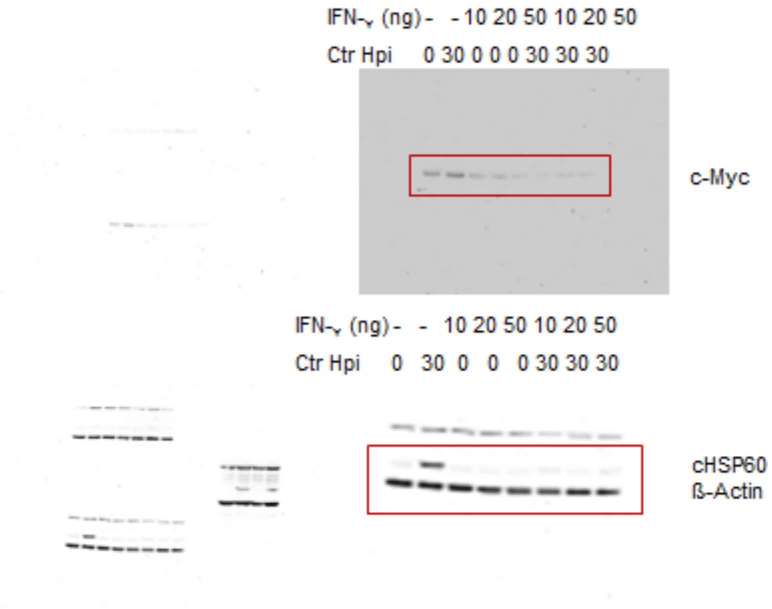

Figure 1 – figure supplement 1 F

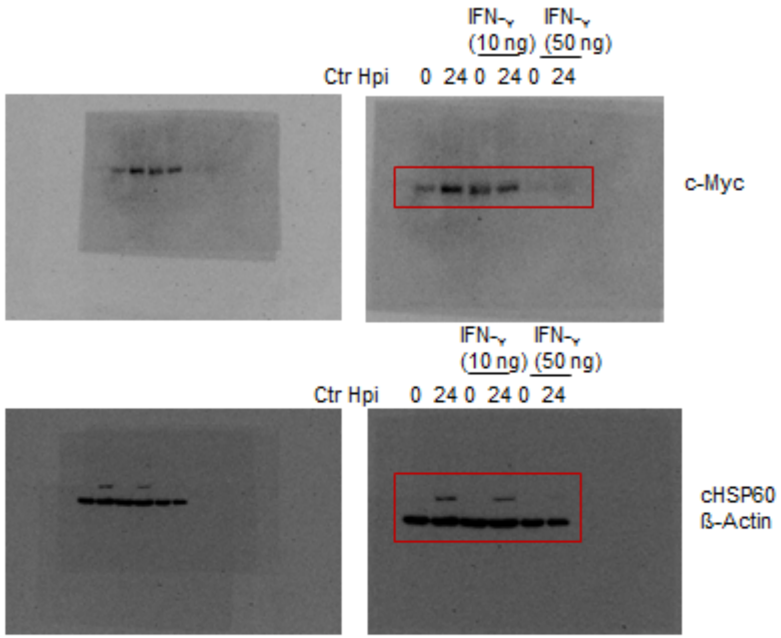

Figure 1 – figure supplement 1 G

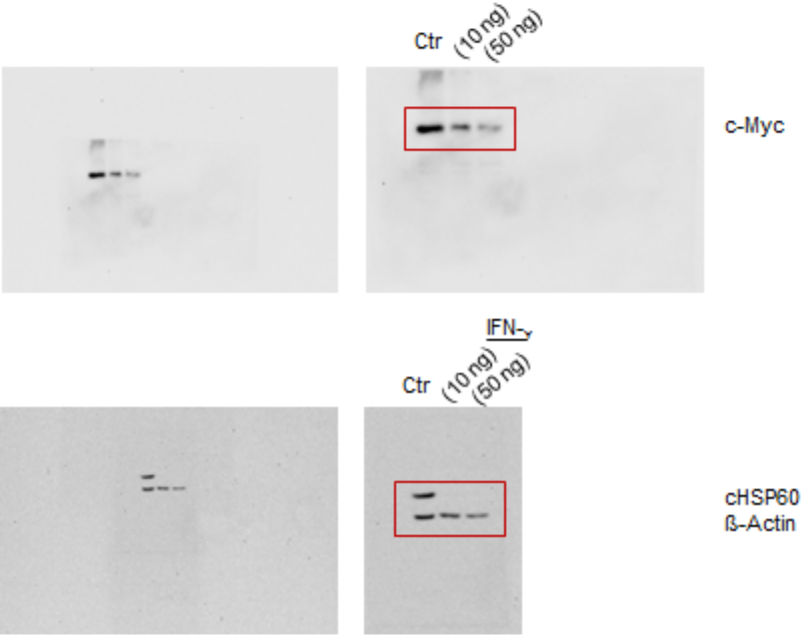

Figure 1 – figure supplement 1 I

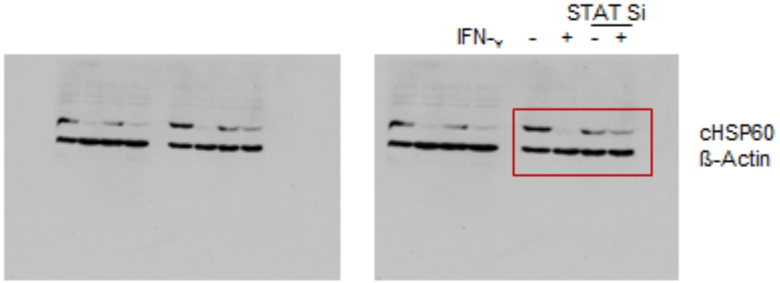

Supplement: Figure 1—figure supplement 1—source data 1. [file elife-76721-fig1-figsupp1-data1.pdf]
