# Supplementary material for: c-Myc plays a key role in IFN-γ-induced persistence of Chlamydia trachomatis
Source: eLife. 2022 Sep 26;11:e76721. doi: 10.7554/eLife.76721 (PMC9512400; doi:10.7554/eLife.76721)

Figure 3 – figure supplement 1 A

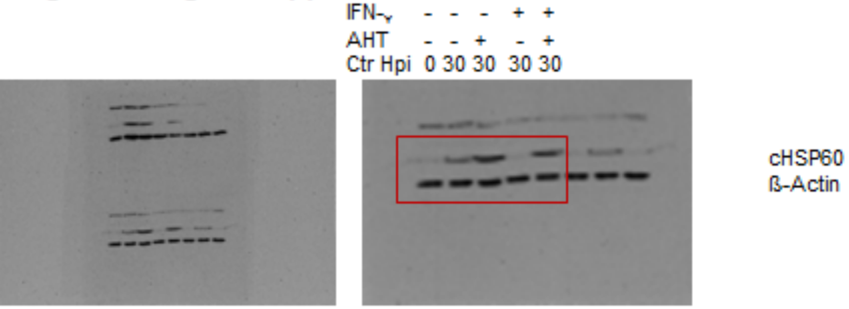

Figure 3 – figure supplement 1 C

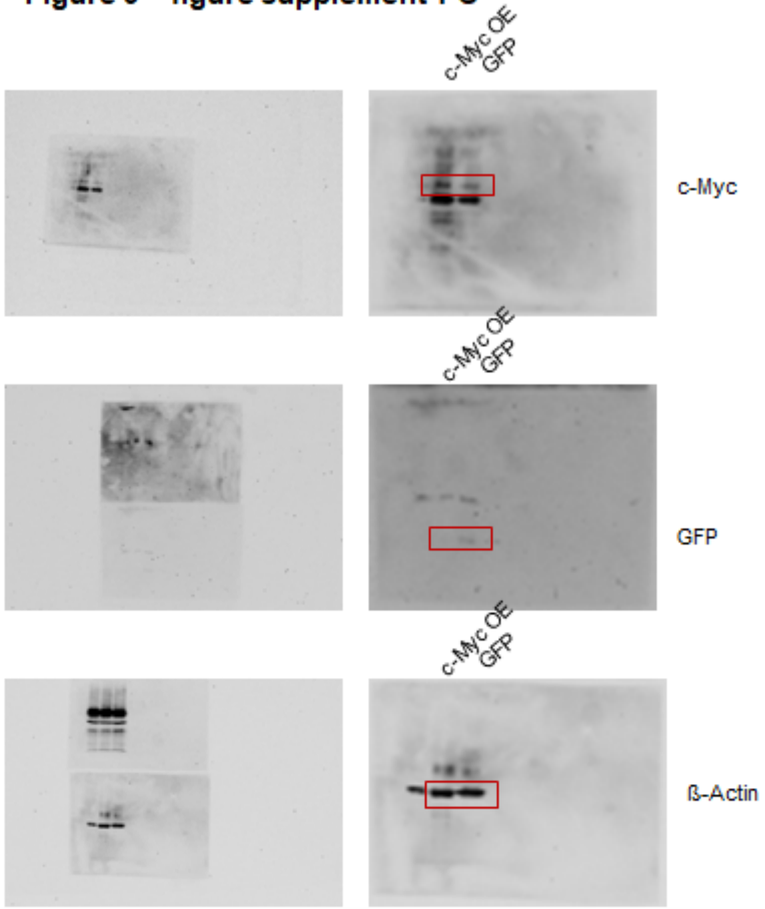

Figure 3 – figure supplement 1 D

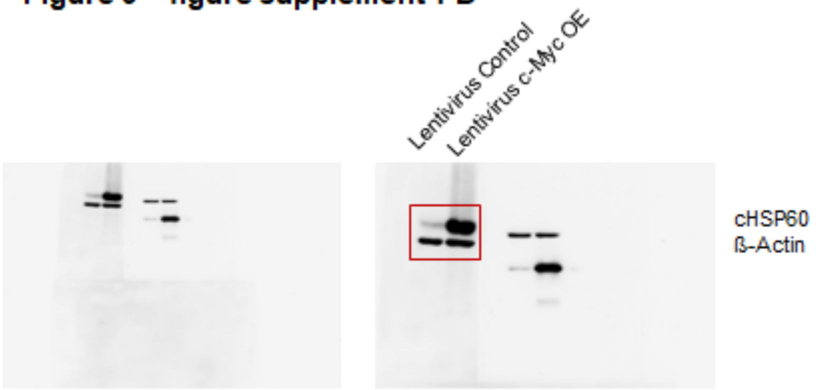

Figure 3 – figure supplement 1 E

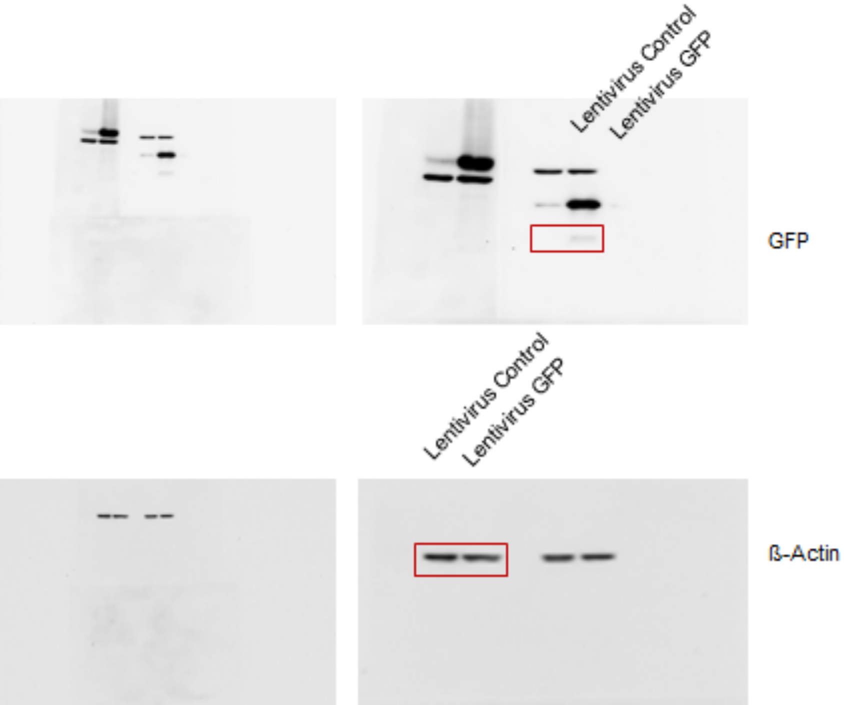

Supplement: Figure 3—figure supplement 1—source data 1. [file elife-76721-fig3-figsupp1-data1.pdf]
