# Supplementary material for: c-Myc plays a key role in IFN-γ-induced persistence of Chlamydia trachomatis
Source: eLife. 2022 Sep 26;11:e76721. doi: 10.7554/eLife.76721 (PMC9512400; doi:10.7554/eLife.76721)

Figure 4 – figure supplement 1 A

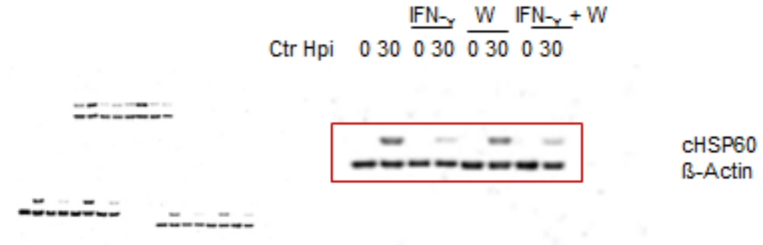

Figure 4 – figure supplement 1 B

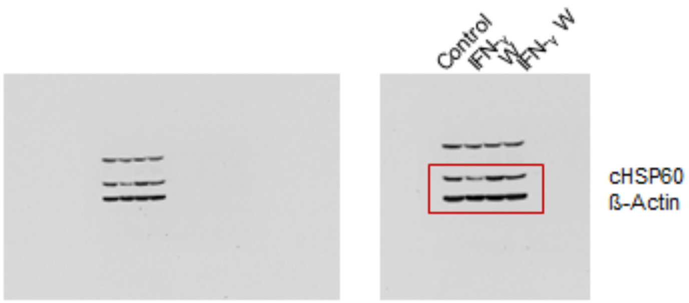

Figure 4 – figure supplement 1 D

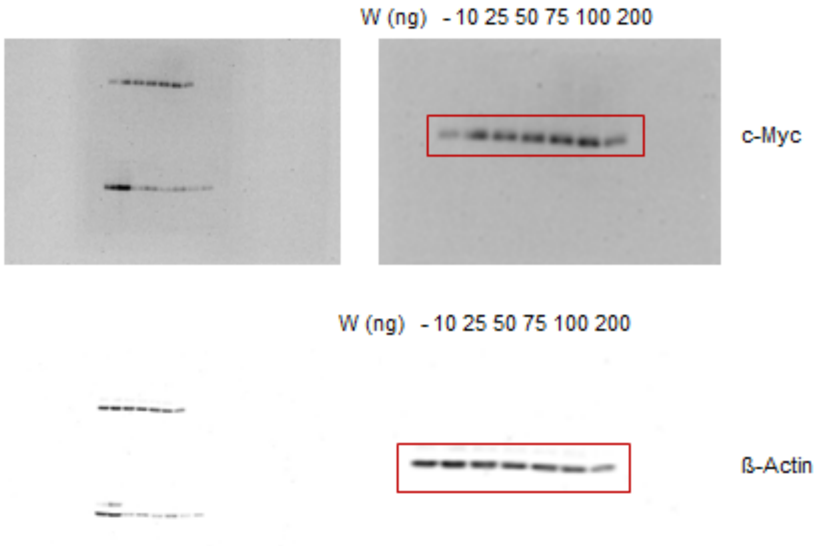

Figure 4 – figure supplement 1 E

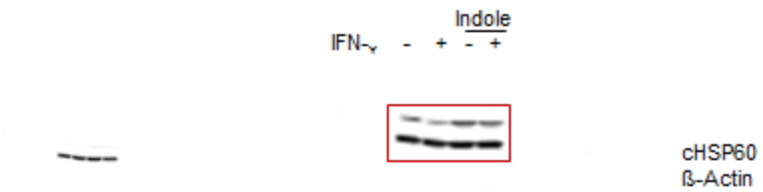

Figure 4 – figure supplement 1 G

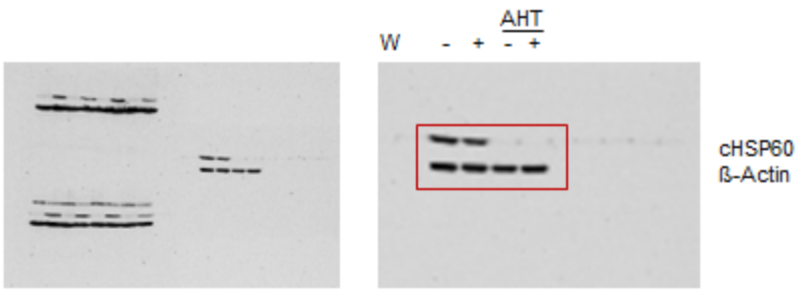

Figure 4 – figure supplement 1 H

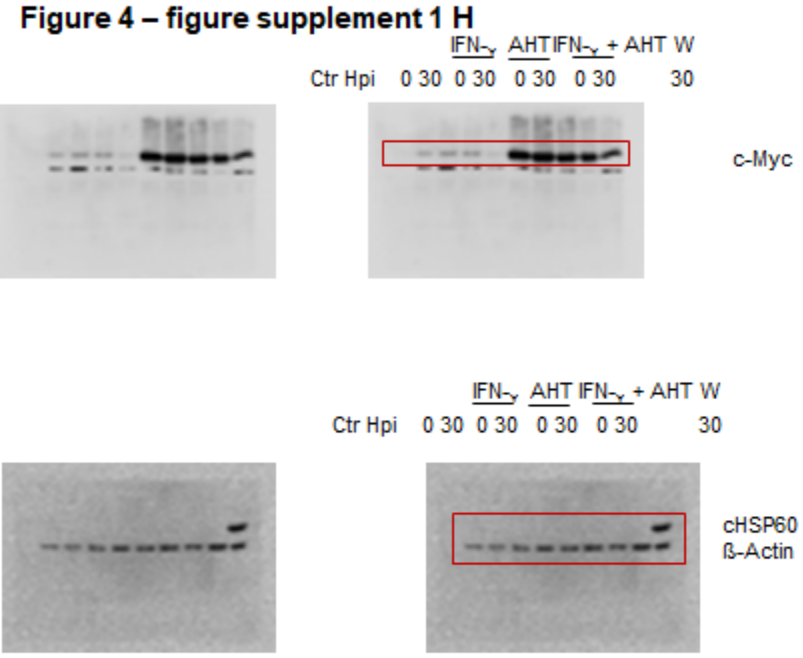

Figure 4 – figure supplement 1 I

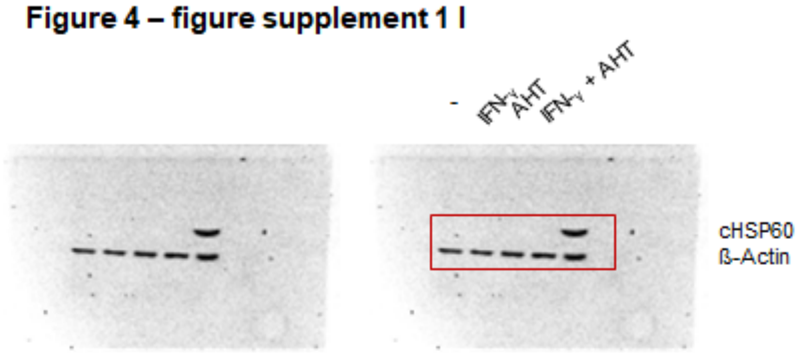

Figure 4 – figure supplement 1 J

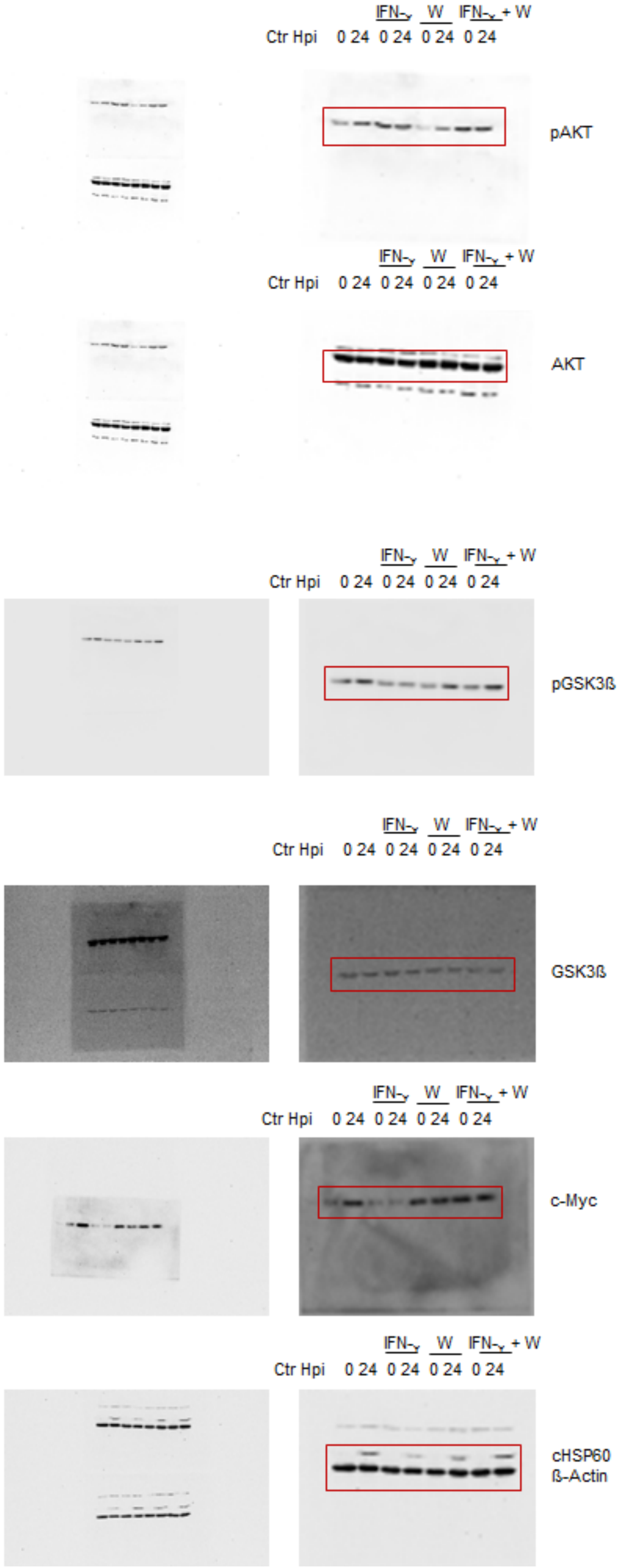

Supplement: Figure 4—figure supplement 1—source data 1. [file elife-76721-fig4-figsupp1-data1.pdf]
